# Nicotine-Mediated Regulation of Nicotinic Acetylcholine Receptors in Non-Small Cell Lung Adenocarcinoma by E2F1 and STAT1 Transcription Factors

**DOI:** 10.1371/journal.pone.0156451

**Published:** 2016-05-26

**Authors:** Courtney Schaal, Srikumar Chellappan

**Affiliations:** 1 Department of Tumor Biology, H. Lee Moffitt Cancer Center and Research Institute, Tampa, Florida, United States of America; 2 Cancer Biology PhD Program, Department of Cell Biology, Microbiology, and Molecular Biology, University of South Florida, Tampa, Florida, United States of America; Roswell Park Cancer Institute, UNITED STATES

## Abstract

Cigarette smoking is the major risk factor for non-small cell lung cancer (NSCLC), which accounts for 80% of all lung cancers. Nicotine, the addictive component of tobacco smoke, can induce proliferation, migration, invasion, epithelial-mesenchymal transition (EMT), angiogenesis, and survival in NSCLC cell lines, as well as growth and metastasis of NSCLC in mice. This nicotine-mediated tumor progression is facilitated through activation of nicotinic acetylcholine receptors (nAChRs), specifically the α7 subunit; however, how the α7 nAChR gene is regulated in lung adenocarcinoma is not fully clear. Here we demonstrate that the α7 nAChR gene promoter is differentially regulated by E2F and STAT transcription factors through a competitive interplay; E2F1 induces the promoter, while STAT transcription factors repress it by binding to an overlapping site at a region -294 through -463bp upstream of the transcription start site. Treatment of cells with nicotine induced the mRNA and protein levels of α7 nAChR; this could be abrogated by treatment with inhibitors targeting Src, PI3K, MEK, α7 nAChR, CDK4/6 or a disruptor of the Rb-Raf-1 interaction. Further, nicotine–mediated induction of α7 nAChR was reduced when E2F1 was depleted and in contrast elevated when STAT1 was depleted by siRNAs. Interestingly, extracts from e-cigarettes, which have recently emerged as healthier alternatives to traditional cigarette smoking, can also induce α7 nAChR expression in a manner similar to nicotine. These results suggest an autoregulatory feed-forward loop that induces the levels of α7 nAChR upon exposure to nicotine, which enhances the strength of the signal. It can be imagined that such an induction of α7 nAChR contributes to the tumor-promoting functions of nicotine.

## Introduction

Lung cancer is the leading cause of cancer-related deaths for both men and women in the United States and worldwide, and accounts for more deaths than breast, prostate, and colon cancers combined [1, 2]. Non-small cell lung cancer (NSCLC) comprises the majority of all lung cancer cases at 85%, and is largely attributable to cigarette smoking which accounts for 80–90% of all lung cancer deaths [3]. Tobacco smoke contains multiple classes of carcinogens including the tobacco specific nicotine derivatives N-Nitrosonornicotine (NNN) and 4-(methylnitrosamino)-1-(3-pyridyl)-1-butanone (NNK), which induce the formation of DNA adducts resulting in mutations of vital genes such as *KRAS*, *Rb*, and *TP53* ultimately leading to tumorigenesis [3, 4] [5]. Nicotine, the addictive component of cigarette smoke, while not typically thought to be carcinogenic, has been shown to induce the proliferation, migration, invasion, and survival of cells from multiple cancer types such as those of the lung, pancreas, bladder, head and neck, as well as gliomas [6–16] indicating its ability to act as a potent tumor promoter. Supporting this contention, nicotine has been shown to promote the growth and metastasis of NSCLC as well as pancreatic cancer in mouse xenograft models when administered via intraperitoneal injection or transdermal patches [9, 17–19]. The primary mechanism by which nicotine exerts these tumor promoting functions is through activation of nicotinic acetylcholine receptors (nAChR) [11, 20–23], which might activate other receptors or directly enhance downstream signaling events.

nAChRs are comprised of pentameric subunits that span the plasma membrane, and are typically expressed at neuromuscular junctions as well as on neuronal cells where they function as ligand-gated ion channels facilitating calcium influx and release of neurotransmitters, inducing multiple signaling cascades [24]. These receptors are also expressed on primary and transformed cells of epithelial and endothelial origin, where they mediate the synthesis and release of neurotrophic factors, growth factors, and proangiogenic factors such as VEGF [22, 25, 26]. While acetylcholine (Ach) is the physiological ligand of nAChRs, nicotine binds to these receptors with greater affinity than Ach and can displace Ach, stimulating a number of tumor promoting signaling cascades [23, 25].

Genome-wide association studies (GWAS) have identified a susceptibility locus for human lung cancer at chromosome 15q24-25, which contains *CHRNA3*, *CHRNA5*, and *CHRNB4* genes encoding the α3, α5, and β4 subunits of nAChRs [27–29]. Polymorphisms in this region were found to correlate with nicotine dependence, number of cigarettes smoked per day, and increased risk for lung cancer development [29]. The α5 subunit has been implicated in smoking-related lung cancer, implicated as the primary central nervous system receptor involved in smoking addiction and behavioral patterns, and additionally has been strongly associated with increased lung cancer risk via a nonsynonymous variation in *CHRNA5 D398N* [27, 30, 31]. Methylation status of CHRNB4 has prognostic value for NSCLC, as demethylation correlates with tumor progression and poor survival in patients with this disease [32]. While multiple nAChRs have been found to be expressed on non-neuronal and NSCLC cells, nicotine-mediated tumor progression is facilitated predominantly through the α7 subunit [11, 17, 25, 33–35]. Consistent with this, α7 levels are found to be elevated in mice that are administered nicotine, and nicotine-mediated effects on cell proliferation, invasion, migration and angiogenic tubule formation are abrogated in the presence of the α7-specific inhibitors [9, 17, 36]. Given this information, study of nAChRs and their regulation in tumor progression is warranted. While α7 nAChR regulation has been reported to be mediated through Sp1/GATA pathway in squamous cell carcinomas of the lung (33), not much information is available on its regulation by other transcription factors or in lung adenocarcinoma. Since nicotine is known to activate E2F transcriptional activity, we sought to elucidate whether E2F as well as other transcription factors play a role in the regulation of α7 in NSCLC cells, in response to exposure to nicotine.

Mitogenic signaling cascades are aberrantly altered in tumors due to mutations or over activation of upstream receptors [37]. Our lab has previously shown that in NSCLC, stimulation of α7 nAChR with nicotine results in the activation Src kinase through the recruitment of the β-arrestin-1 scaffolding protein, followed by the subsequent activation of Raf-1 kinase which phosphorylates Rb tumor suppressor protein [34, 35, 38]. Upon phosphorylation by Raf-1 and CDK/cyclins, Rb becomes hyperphosphorylated and dissociates from E2F transcription factors allowing them to activate expression of E2F target genes, including those involved in cell cycle and tumor progression [11, 34]. Upon nicotine stimulation the β-arrestin-1 scaffolding protein itself can translocate to the nucleus where it binds to E2F transcription factors and increases expression of target genes [35, 38]. The studies presented here show how nicotine upregulates the levels of the α7 nAChR subunit through the involvement of E2F1 transcription factor, which suggests the existence of a feed-forward mechanism by which the downstream signals mediated by nicotine might be amplified.

## Materials and Methods

### Kaplan Meier Survival Plots

The Kaplan Meier survival curves were generated using KM-plotter online analysis tool (http://kmplot.com/analysis), which has previously been described in detail [39–41]. KM-plotter is a meta-analysis based method of assessing biomarkers in breast, ovarian, lung, and gastric cancers using data derived from publicly available microarray databases GEO (Gene Expression Omnibus), TCGA (The Cancer Genome Atlas), and EGA (European Genome-phenome Atlas). To date, it is capable of assessing the effect of 54,675 Affymetrix gene-chips on survival outcome using 10,888 patient samples; including 2,437 lung cancer cases. For our analysis, parameters were set using the 2015 version of the analysis tool, univariate cox regression analysis was used, and biased arrays were excluded. Gene symbols CHRNA7, CHRNA3, and CHRNA5 were assessed in NSCLC for all histological subtypes, smoking status, and gender differences. Gene expression which correlated with survival with a *p* value <0.05 was considered statistically significant.

### Cell Culture and Reagents

Human non-small cell lung adenocarcinoma cell lines A549 and H460 were obtained from the American Type Culture Collection (ATCC, Manassass, VA). A549 cells were maintained in Ham’s F12K medium (Cellgro, Mediatech, Inc. Manasses, VA) supplemented with 10% fetal bovine serum (Atlas Biologicals, Fort Collins, CO), and H460 cells were maintained in RPMI 1640 (Gibco, Life Technologies, Thermo Fisher Scientific Inc., Waltham, MA) containing 10% fetal bovine serum.

For studies using nicotine (Sigma-Aldrich) or e-cigarettes Fin, NJoy, Mistic (local stores), cells were rendered quiescent by serum starvation for 24 hours, following which cells were stimulated with nicotine or e-cigarette extracts for the indicated time points. For studies using signal transduction inhibitors/anti-cancer drugs, cells were rendered quiescent by serum starvation for 24 hours, were treated with inhibitors for 30 minutes, and then stimulated with 2μM nicotine in the presence or absence of inhibitors for 48 hours. The inhibitors used were AZD0530/Saracatinib at 10μM, NVP-BKM120/Buparsilib at 20μM, GSK1120212/Trametinib at 10μM, LEE001/Ribociclib at 20μM, RRD251 at 10μM, and α-bungarotoxin at 10μM.

For our studies, e-cigarette liquid was obtained from the cartridge through extraction of an internal liquid-soaked sponge within the device for Fin and NJoy brands, or through syringe extraction for Mistic brand. Fin, NJoy, and Mistic brand extracts were 1.6% nicotine by volume (NBV) or 16mg/ml, 1.5% NBV or 15mg/ml, and 1.8% NBV or 18mg/ml respectively as indicated on the manufacturer’s packaging. Molarity of extracts from each brand was calculated based on the molecular weight of nicotine of 162.23, and the working concentration of 1.5uM was achieved by serial dilutions of 1:10, 1:9, or 1:11 for Fin, NJoy, and Mistic respectively, to achieve 10mM, then diluted 1:100 to achieve 100uM, and diluted 1:75 for a final concentration of 1.5uM.

### ChIP Assays

ChIP assays were conducted on asynchronous A549 and H460 cell lines as previously described [42]. ChIP lysates were incubated with 25ug/ml of the following antibodies: E2F1 rabbit polyclonal (sc193), E2F2 rabbit polyclonal (sc633), E2F3 rabbit polyclonal (sc879), E2F4 rabbit polyclonal (sc1082), E2F5 rabbit polyclonal (sc999), Rb rabbit polyclonal (sc50), STAT1 p84/p91 rabbit polyclonal (sc346), and STAT3 mouse monoclonal (sc8109) which were purchased from Santa Cruz Biotechnologies. A rabbit anti-mouse secondary antibody from Pierce was used as a negative control IgG. Interactions of the proteins with specific regions of the α7 promoter were detected by polymerase chain reaction (PCR) amplification using the following primer sequences: α7.1 forward 5’-TCGGGTCTGTTTTGTCTGGTT-3’ and α7.1 reverse 5’CAGAAGCTGCGCTGGGCACTC-3’ spanning a 147bp region at position -317 through -464bp upstream of the promoter’s TSS; and α7.2 forward 5’-GTACCCAGCGCCGGGAGTAC-3’ and α7.2 reverse 5’-GCTCGCGCGCCTTTAAGGAG-3’ spanning an 189bp region at position +15 through -174 where TSS = 0. PCR results were quantitated using ImageJ software and are represented as percent of input control as graphical data.

### Transient Transfections and Luciferase Assays

A549 or H460 cells were cultured to 70% confluency and transfected in Opti-MEM medium (Gibco, Life Technologies) using Fugene HD (Promega) transfection reagent following the manufacturer’s protocol. The 235bp and 1115bp α7-luciferase gene promoter constructs were kindly provided by Dr. Sherry Leonard (University of Colorado, Denver, CO). The expression vectors used were pcDNA3-HA-E2F1, pcDNA3-E2F2, pcDNA3-E2F3, pcDNA3-E2F4, pcDNA3-E2F5, pcDNA3-STAT1, and pcDNA3-STAT3. Empty vector pcDNA3 was used as a control. Luciferase assays were conducted 24 to 48 hours after transfection per manufacturer’s protocol using the Dual Luciferase Assay system (Promega). Results are reported in terms of relative luciferase activity (RLA) based on the ratio of RLUs1 firefly luciferase to RLUs2 Renilla luciferase (normalization control) values as measured on a Turner Biosystems luminometer.

### siRNA Transfections and Quantitative Real-Time PCR

A549 or H460 cells were grown to 70% confluency and transfected in Opti-MEM (Gibco Life Technologies) with 100pmol of siRNAs for E2F1 (sc29297) and STAT1 (sc44123) (Santa Cruz Biotechnologies) using Oligofectamine reagent (Invitrogen) per manufacturer’s protocol. 4–6 hours after transfection, media was replaced by complete medium containing 10% FBS. RNA was isolated using Qiagen RNEasy miniprep kit (Hilden, Germany) according to manufacturer’s protocol. First strand cDNA was synthesized using Bio-Rad iScript cDNA synthesis kit (Hercules, CA). mRNA expression was assessed using quantitative real-time PCR (qRT-PCR) (Bio-Rad CFX96 Real Time System) and data were analyzed using the CFX96 software programming and the ΔΔCT method. GAPDH levels were used for normalization. RT-primers used were as follows: α7 forward 5’-TCCTGCACGTGTCCCT-3’ and α7 reverse 5’-CTTGGTTCTTCTCATCCACG-3’ at 55 degrees Celsius; and GapDH forward 5’-GGTGGTCTCCTCTGACTTCAACA-3’ and GapDH reverse 5’-GTTGCTGTAGCCAAATTCGTTGT-3’.

### Lysate Preparation and Western Blot Analysis

Cells were washed twice with cold 1xPBS, scraped off the plates, collected by centrifugation for 5 minutes at 6,000rpm, and lysed in M2 lysis buffer (20mM Tris-HCl pH6.0, 0.5% NP-40, 250mM NaCl, 3mM EGTA, and 3mM EDTA) containing protease inhibitors as described in our previous work [38]. After lysis, protein concentration was measured using Bradford assay (BioRad) and equal amounts of proteins were resolved on 8% SDS-page polyacrylamide gels, and transferred onto nictrocellulose membranes using BioRad semi-dry transfer unit. Membranes were blocked using 5% nonfat dry milk in 1xPBS containing 0.1–0.5% Tween20. After rinsing with 1xPBST, membranes were incubated overnight at 4 degrees Celsius with 2.5μg/mL α7 nAChR rabbit polyclonal (Abcam ab23832 and ab10096) or 1:25,000 β-actin mouse monoclonal (Sigma-Aldrich A1978) primary antibodies, washed again in 1xPBST, incubated for one hour at room temperature in HRP-conjugated secondary antibodies at 1:3000 dilutions, and protein was detected using ECL reagent from GE Healthcare or Pierce Biotechnology according to standard protocols. Results were quantitated using ImageJ software where protein expression was normalized to the corresponding β-actin, and represented as fold change relative to the control as graphical data.

### In-cell Western Blots

A549 cells were washed three times with PBS, fixed for 20 minutes using 10% buffered formalin, permeabilized using 0.2% Triton-x, blocked for 1 hour at room temperature, and incubated in primary antibody overnight. α7 rabbit antibody (Abcam ab10096) was used at 2.5μg/ml and mouse β-actin antibody (Sigma) was used at 5.0μg/ml. Following primary antibody incubation, cells were incubated in rabbit 700cw IRDye (Li-Cor) and mouse 800cw (Li-Cor) secondary antibodies for 1 hour at room temperature, washed with 1xPBS, and imaged using Li-Cor Odyssey 9120 Infrared Imaging System. Quantitative analysis was conducted using the Odyssey analysis software.

### Immunofluorescence Analysis and Confocal Microscopy

A549 cells were plated onto poly-D-lysine coated 8-well glass chamber slides (LabTek), washed three times with PBS, fixed for 20 minutes at room temperature in 10% buffered formalin, permeabilized using 0.2% Triton-x, blocked for one hour in 5% goat serum, and incubated overnight in primary antibody α7 nAChR rabbit antibody (Abcam ab10096) at a concentration of 2.5μg/ml. Following primary antibody, cells were incubated in anti-rabbit Alexa Fluor-488 secondary antibody (Life Technologies) for 1 hour, and mounted using DAPI Vectashield (Vector Labratories). Cells were visualized with a DM16000 inverted Leica TCS SP5 tandem scanning confocal microscope.

## Results

### Levels of α3, α5, and α7 nAChRs correlate with lung cancer patient survival

**Fig 1:**
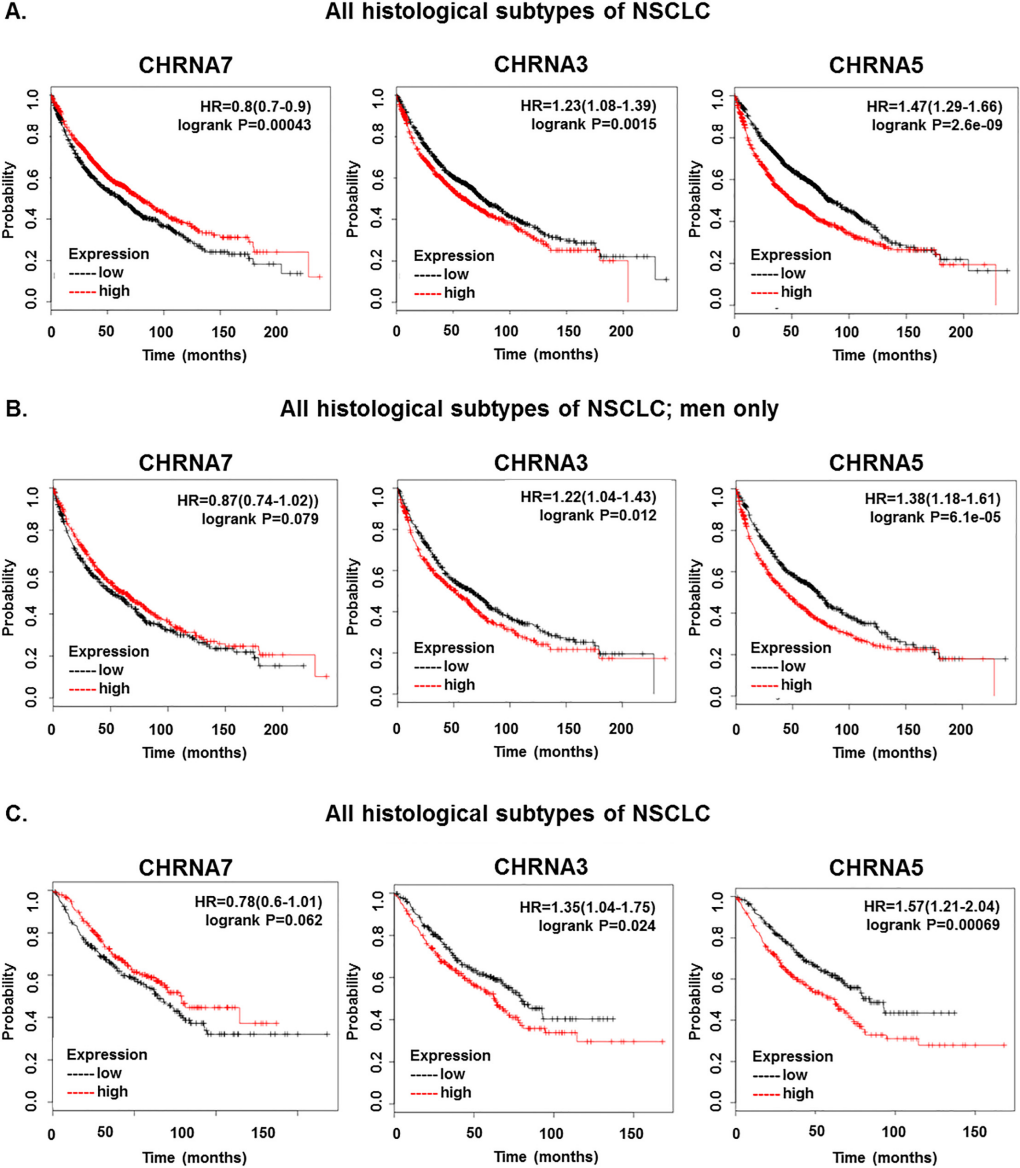
CHRNA7, CHRNA3, and CHRNA5 Correlate with Patient Survival in NSCLC. (A) Association of CHRNA7, CHRNA3, and CHRNA5 expression with survival outcome across all histological subtypes of NSCLC. (B) Association of CHRNA7, CHRNA3, and CHRNA5 with survival outcome across all histological subtypes of NSCLC in men only. (C) Association of CHRNA7, CHRNA3, and CHRNA5 with survival outcome across all histological subtypes of NSCLC in men who smoke.

The Kaplan-Meier plotter web-based tool available on KMplot.com was used to analyze a database of 2,437 NSCLC patient cases [39] to assess whether patient survival outcome correlated with expression of the α7 nAChR subunit which is functionally implicated in lung cancer progression, or α3 and α5 nAChR subunits which were identified to correlate with lung cancer incidence in GWAS studies [27–29]. High expression of α7 correlated with increased survival across all histological subtypes and variants of NSCLC cases, with a *p* value of 0.00043. High expression of α3 significantly correlated with decreased survival probability in all histological subtypes and variants of NSCLC, with a *p* value of 0.0015. Similarly, high levels of α5 correlated with decreased survival probability across all histological subtypes and variants of NSCLC with a *p* value of 2.6e-09 (Fig 1A). When survival outcome and expression of α7, α3, and α5 were analyzed for gender criteria, we found that high levels of α7 expression correlated with increased survival while high levels of α3 and α5 expression correlated with decreased survival in men (*p*-values = 0.079, 0.012, and 6.1e-05 respectively); surprisingly, no correlation was found in women (Fig 1B). NSCLC frequently occurs in patients who are current or former smokers, and nAChRs are implicated in smoking-related NSCLC progression [43], so we next analyzed correlation of survival outcome and α7, α3, and α5 expression in male patients who were smokers. High α7 levels correlated with increased survival in male smokers, while high levels of α3 and α5 correlated with decreased survival outcome in male patients who smoked (*p*-value = 0.00043, 0.024, and 0.00069 respectively)(Fig 1C). No differences were seen when histological subtype or stage were analyzed.

Collectively, these data show that high levels of α7 expression correlates with increased survival probability, which was unexpected, while α3 and α5 expression correlate with decreased survival probability across all histological subtypes of lung cancer, in men, and in men excluding never smokers. Here we report that E2F and STAT transcription factors regulate α7 nAChR gene expression.

### E2F and STAT transcription factors regulate α7 nAChR gene expression

**Fig 2:**
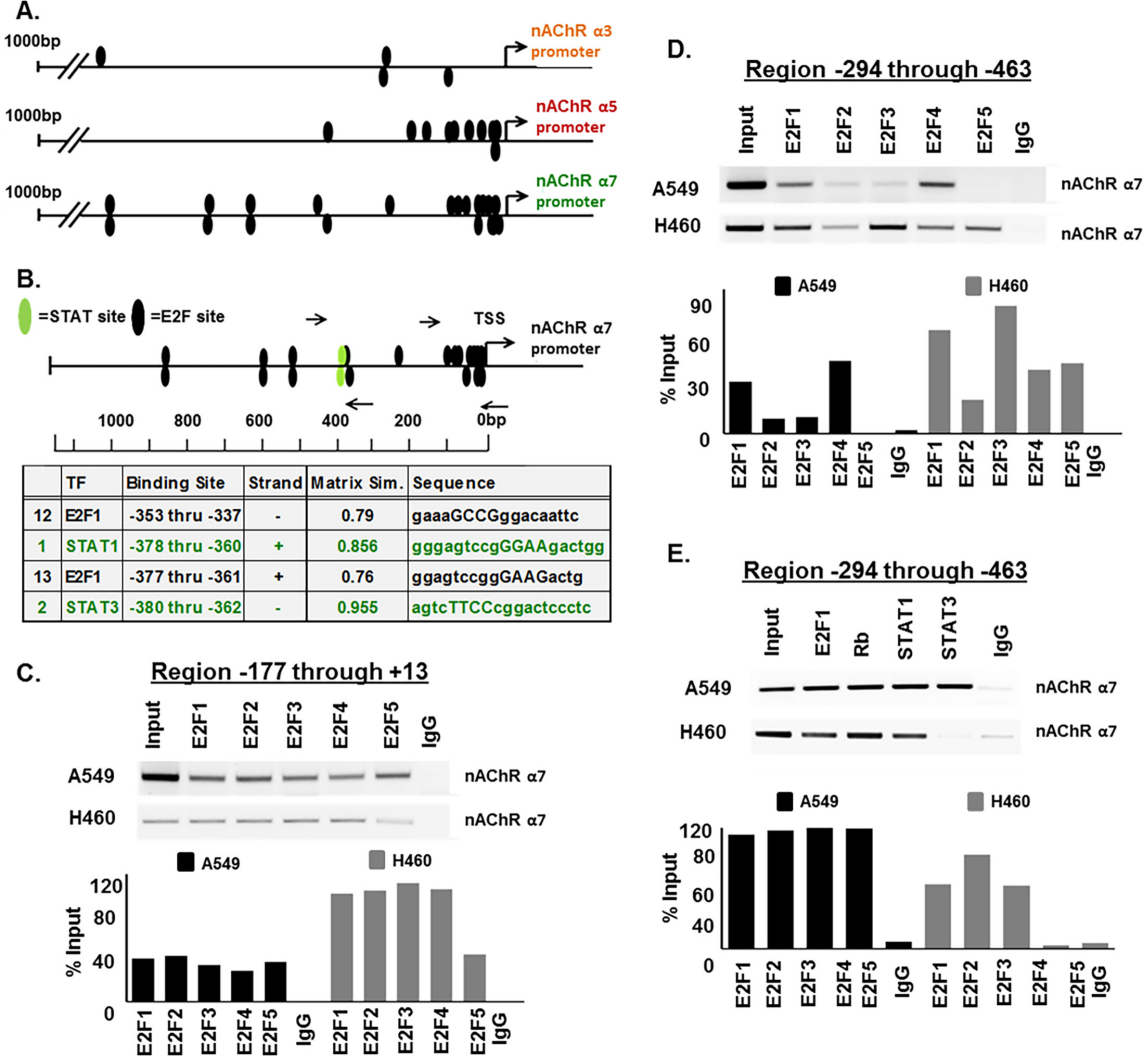
E2F and STAT Transcription Factors Have Predicted Binding Sites and can Bind to the α7 Promoter. (A) Schematic representation of 1000bp regions of the α3 nAChR, α5 nAChR, and α7nAChR gene promoters showing potential E2F binding sites as black ovals. (B) Schematic representation of 1100bp region of nAChR α7 gene promoter showing potential E2F binding sites as black ovals and potential STAT binding sites as green ovals. Arrows represent the position of primers spanning E2F and STAT binding sites used for ChIP assays. Sequence data of overlapping E2F and STAT binding sites is detailed. (C) ChIP assays showed binding of E2F1-5 to the α7 nAChR promoter region -177 through +13 in A549 cells and binding of E2F1-4 in H460 cells. E2F1 and E2F4, and to some extent E2F2 and E2F3 could also bind region -294 through -463 in A549 cells while E2F1-5 could bind this region in H460cells. STAT1 and STAT3 additionally could bind region -294 through -463 in A549 cells, while STAT1 alone could bind in H460 cells. Sonicated DNA was used as input control, and there was no detectable amplification from irrelevant IgG, used as negative control. Quantification of the data is depicted as percent of input, in the corresponding graphs.

We first sought to determine whether E2F transcription factors were involved in the activation of the promoters of the α3, α5, and α7 genes, since these subunits have been implicated in smoking related lung cancer and E2Fs are activated downstream of nAChRs in response to nicotine stimulation [27–29]. Genomatix MatInspector analysis of 1000 base pair (bp) regions upstream of the transcription start sites (TSS) of each promoter revealed multiple putative E2F binding sites including four sites on the α3 promoter, ten sites on the α5 promoter, and nineteen sites on the α7 promoter (Fig 2A). During analysis of the α7 promoter, it was noted that there were also two predicted STAT transcription factor binding sites which overlapped with two of the predicted E2F sites at regions -337 through -380bp upstream of the TSS (Fig 2B). This was of interest as we have previously found that nicotine stimulation could induce STAT activation thereby activating a number of STAT target genes involved in tumor progression. Since α7 is the primary subunit facilitating nicotine-mediated tumor progression and was found to contain the greatest number of predicted E2F binding sites, we focused our studies primarily on this subunit.

**Fig 3:**
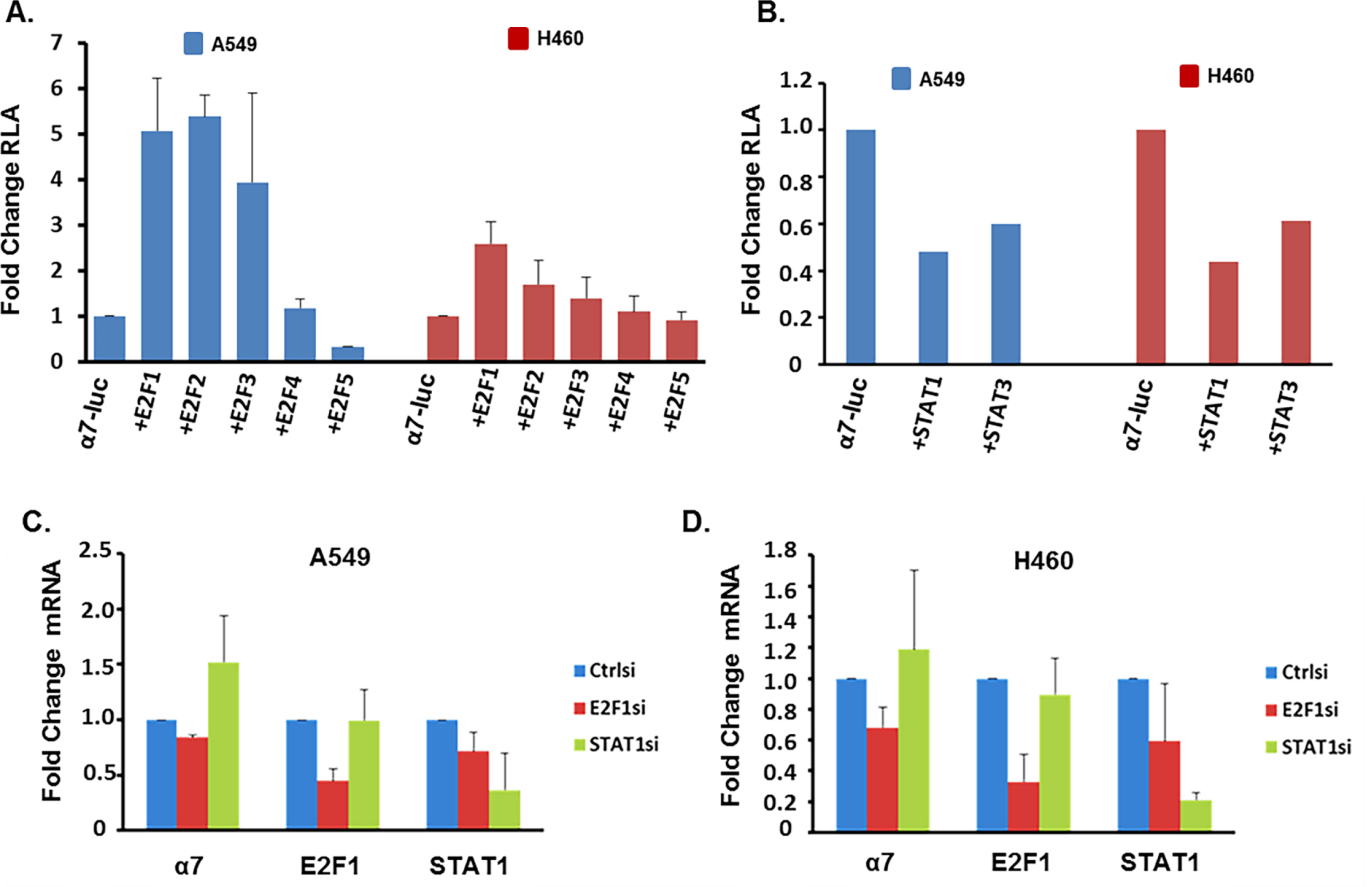
E2F and STAT Transcription Factors Regulate α7 nAChR Expression. (A and B) Transient transfection experiments showing that E2F1-3 can induce the α7 promoter, E2F4 has no effect, and E2F5, STAT1, and STAT3 act repress it in A549 and H460 cells. (C and D) Depletion of E2F1 by siRNA results in reduced expression of α7 mRNA, while depletion of STAT1 results in increased expression in A549 and H460 cells.

To validate whether E2F and STAT transcription factors could bind to the α7 promoter, chromatin immunoprecipitation (ChIP) assays were conducted on A549 and H460 cells, both of which harbor K-Ras mutations, which frequently occur in lung cancer patients who smoke. It was found that E2F1, E2F2, E2F3, E2F4, and E2F5 could bind at region -177 through +13bp upstream of TSS, where 10 predicted E2F sites were clustered on the promoter, in both A549 and H460 cell lines (Fig 2C); quantification of the bands are shown in the bottom panel. E2F1 and E2F4 additionally were shown to bind at region -294 through -463bp upstream of the TSS in A549 where the two predicted E2F sites overlap with the two predicted STAT sites; and E2F1, E2F2, E2F3, E2F4, and E2F5 were shown to bind this site in H460 (Fig 2D); the bands are quantified in the lower panel. STAT1 and STAT3 also bound the promoter at region -294 through -463bp upstream of the TSS where the two predicted STAT sites overlap with two E2F sites in A549; STAT1 but not STAT3 bound to this region in H460s (Fig 2E); the results are quantified in the lower panel. Experiments were conducted to determine whether E2F and STAT transcription factors which bound the α7 promoter could regulate its expression. Transient transfection experiments were carried out using an 1115bp α7-luciferase promoter construct which we received from the lab of Sherry Leonard at the University of Colorado, Denver. Co-transfection using 0.5μg of the reporter construct with 1μg of E2F1, E2F2, E2F3, or E2F4 expression vectors resulted in 5.0, 5.4, 3.9, or 1.2 fold induction of α7-luciferase expression respectively; conversely co-transfection with 1μg of E2F5, STAT1, and STAT3 expression vectors resulted in 70%, 50%, and 40% reduction in expression of α7-luciferase reporter in A549 respectively, demonstrating the ability of these proteins to differentially regulate the α7-luciferase promoter (Fig 3A and 3B). Similar results were observed in H460s where co-transfection of α7-luciferase promoter with E2F1, E2F2, or E2F3 resulted in 2.6, 1.7, or 1.4 fold induction respectively; E2F4 had little effect on expression at 1.1 fold induction, E2F5, STAT1, and STAT3 resulted in 10%, 60%, and 40% reduction of expression respectively. To further assess the role of E2F1 and STAT1 in the regulation of α7 promoter, E2F1 or STAT1 were depleted in A549 or H460 cells by transient transfection using 100pmol of small interfering RNAs (siRNA) and expression of α7 mRNA was assessed using qRT-PCR. α7 mRNA was reduced by 20% in A549 or 30% in H460 upon depletion of E2F1, while α7 mRNA levels were increased by 1.5 fold in A549 or 1.2 fold in H460 upon depletion of STAT1, further suggesting that E2F1 induces α7 expression while STAT1 represses it (Fig 3C and 3D).

### E2F1 and STAT1 differentially regulate α7 nAChR

**Fig 4:**
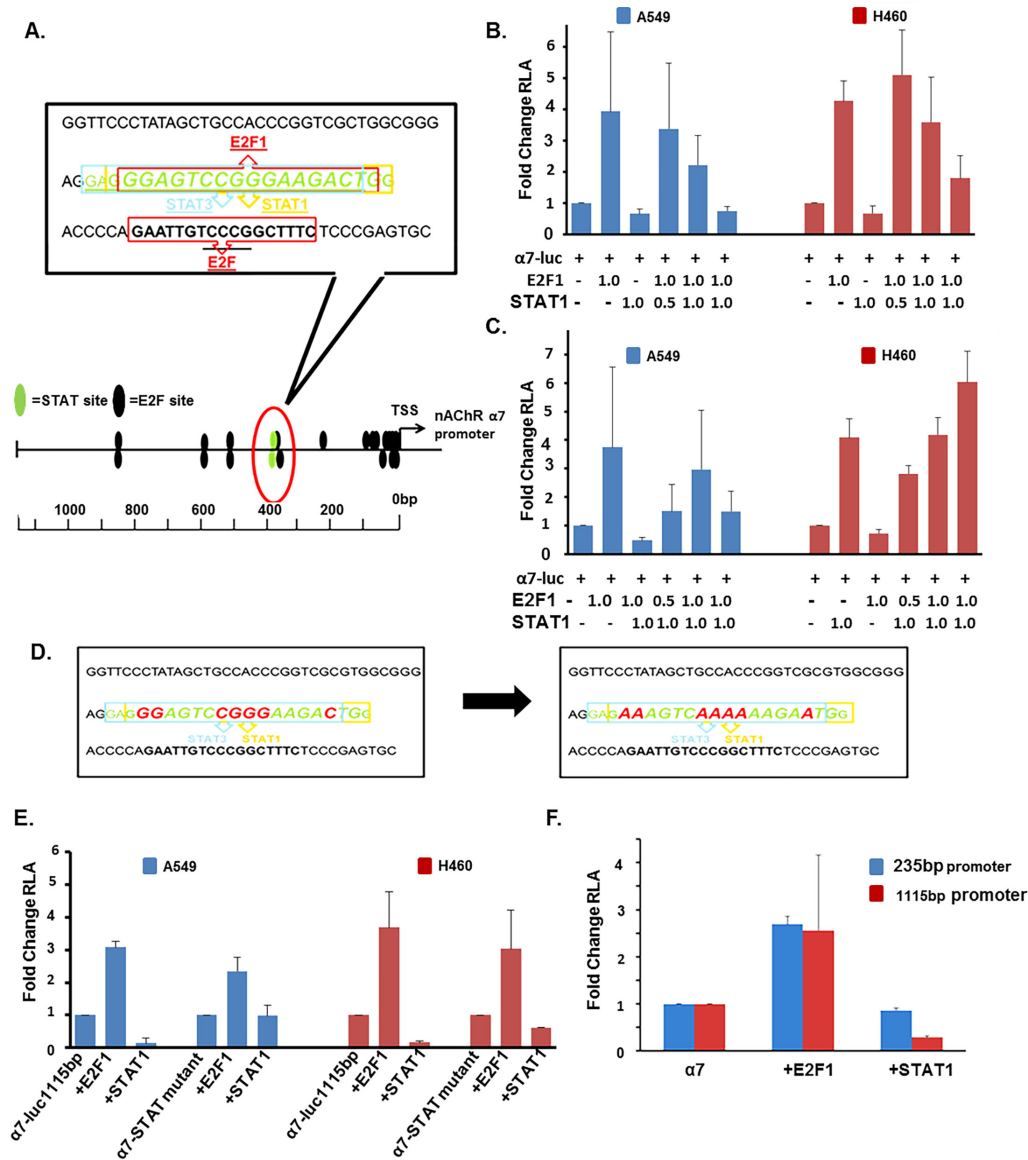
E2F and STAT Have Overlapping Binding Sites on the α7 Promoter, and Differentially Act to Regulate its Expression. (A) Schematic representation of the overlapping E2F1 and STAT1/3 binding sites on the α7 promoter at region -294 through -463. (B and C) Transient transfection showing increasing concentrations of STAT1 can repress E2F1-mediated induction of the α7 promoter in A549 cells; reciprocally increasing concentrations of E2F1 can alleviate STAT1-mediated repression of the α7 promoter in A549 cells. (D) Schematic representation of DNA sequence of overlapping E2F and STAT binding sites. Nucleotides which were mutated to disrupt STAT binding on α7 promoter are depicted in red on the left and the resulting mutated sequence is depicted in red on the right. (E) Transient transfections showed that STAT1 could repress the α7 promoter but not the α7-STAT-site-mutant promoter in A549 and H460 cells. (F) Transient transfection showed that STAT1 could repress the 1115bp α7 promoter, but not 235bp α7 promoter lacking predicted STAT binding sites.

Since E2F1 and STAT1 were found to have the greatest effect on α7 expression, we focused on these two factors for further studies. The two predicted STAT binding sites overlapped with two putative E2F sites in the region -294 through -463bp upstream of the TSS on the α7 promoter; so we next sought to determine whether the differential regulation of α7 expression observed was occurring *via* competitive interplay between E2F1 and STAT1 transcription factors binding to this region (Fig 4A). Transient transfection experiments were conducted using α7-luciferase co-transfected with 1μg of E2F1 and increasing concentrations (0.5μg, 1.0μg, and 2.0μg of STAT1); STAT1 could repress E2F1 mediated induction of α7-luciferase in a dose dependent manner (Fig 4B). Reciprocally, 0.5μg, 1.0μg, and 2.0μg of E2F1 could alleviate repression mediated by 1μg of STAT1 on α7-luciferase expression in a concentration dependent manner (Fig 4C). Thus each transcription factor could abrogate the effect of the other, suggesting a competitive interplay occurring at this region of the α7 promoter.

Additional experiments were conducted to confirm this possibility. Towards this purpose, site directed mutagenesis was conducted. Nucleotides critical for STAT1 binding on the α7-luciferase promoter at position -149, -144, -143, -142, -141, -136, and -135bp upstream of TSS were mutated to disrupt STAT1 binding; these sites coincided with the overlapping E2F binding site (Fig 4D). Transient transfections were conducted using 0.5μg of α7-luciferase or α7-luciferase-STAT-mutant promoter constructs with 1μg of E2F1 and STAT1; STAT1 had diminished ability to repress α7 when the binding site was mutated (Fig 4E). STAT1 reduced α7-luciferase promoter activity by 86% and 83% in A549 and H460 cells respectively, while STAT1 reduced α7-luciferase-STAT-mutant promoter activity by 0.02% and 39% in A549 and H460 cells, respectively. E2F1 could induce α7-luciferase promoter activity 3.1 and 3.7 fold in A549 and H460 cells, and could induce the α7-luciferase-STAT-mutant promoter activity 2.3 and 3.0 fold in A549 and H460 cells, respectively. Further, transient transfections were conducted using 0.5μg of a truncated 235bp α7-promoter construct lacking the two STAT binding sites (but retaining ten E2F binding sites), or the full length 1115bp α7-promoter construct 1μg of STAT1 could repress the 1115bp α7-promoter construct by 70%, while STAT1 resulted in a 10% repression of the 235bp α7-promoter construct. E2F-mediated induction of both the 1115bp and 235bp α7-luciferase promoters remained similar at 2.56 and 2.69 fold induction, respectively (Fig 4F). This data suggest that region -294 through -463bp upstream of the TSS on the α7 promoter is critical for STAT-mediated repression; E2F1 could still induce the promoter, through additional sites close to the TSS.

### Nicotine and E-cigarette extracts enhance α7 nAChR expression

**Fig 5:**
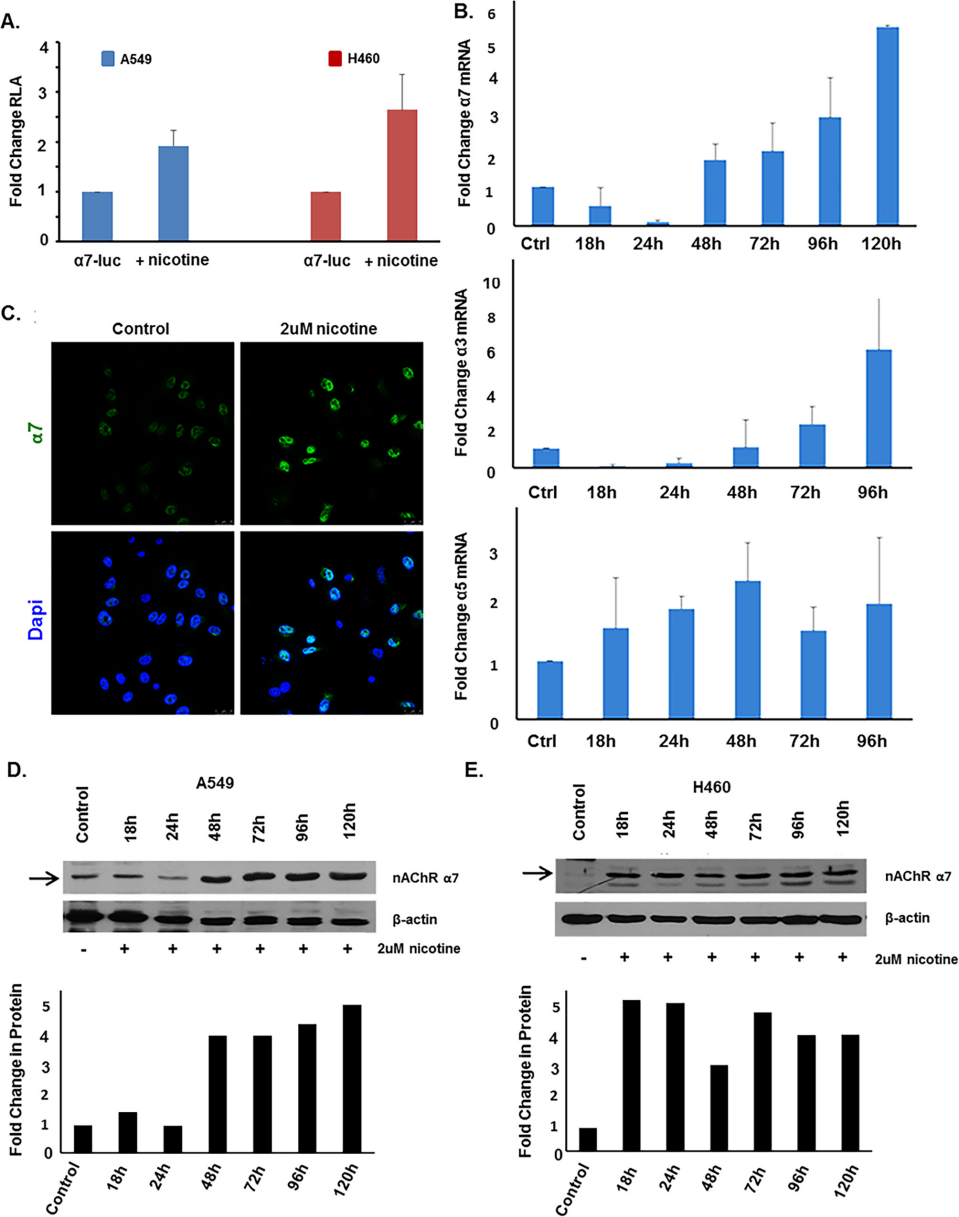
Nicotine can Induce α7 Expression by 48 Hours. (A) Transient transfection showed that 2μM nicotine could induce the α7 promoter after 48 hours of stimulation in A549 cells. (B) qRT-PCR analysis showed that 2μM nicotine induced α7 mRNA levels at 48, 72, 96, and 120 hours, but not at 18 or 24 hours. 2μM nicotine reduced α3 mRNA expression at 18 and 24 hours, little effect was seen at 48hrs, and expression was induced by 72 and 96 hours. 2μM nicotine induced α5 mRNA levels at 18, 24, 48, and 96 hour time points. (C) Immunofluorescent staining showed that 2μM nicotine could induce α7 at the protein level after 48 hours in A549 cells. (D) Western blot analysis showed 2μM nicotine induced α7 protein levels at 48, 72, 96, and 120 hours; an effect not seen at 18 or 24 hour time points in A549 cells. In H460 cells, 2μM nicotine induced α7 protein levels at 18, 24, 48, 72, 96, and 120 hour time points. Arrows indicate the α7 protein band of interest, at approximately 50 kD. Quantification of the western blot data is depicted in the corresponding graphs.

Nicotine has been shown to enhance α7 in adenocarcinoma and squamous cell carcinoma of the lung in vitro as well as in mouse lung adenocarcinoma models [17, 33, 34, 44] Transient transfection experiments were conducted to assess whether nicotine induces the α7 promoter. Towards this purpose, A549 and H460 cells were transiently transfected with 1μg of 1115bp α7-luciferase promoter, serum starved for 24 hours, and stimulated with 2μM nicotine for 24 hours. Upon nicotine stimulation, α7-luciferase activity increased by 1.9 and 2.6 fold in A549 and H460 cell lines respectively, compared to unstimulated cells (Fig 5A). Time course experiments showed that in A549, mRNA levels of α7 decreased by 50% at 18 hours and 90% at 24 hours, but increased at 48, 72, 96 and 120 hours reaching a 5.1 fold increase by 120 hours (Fig 5B, top panel). To determine whether the other nAChRs implicated in lung cancer are also impacted by nicotine, α3 and α5 mRNA levels were additionally assessed by qRT-PCR after 18, 24, 48, 72, or 96 hours of nicotine treatment. α3 was reduced by 92% and 76% at 18 and 24 hours, but induced by 2.25 and 6.1 fold by 72 and 96 hours post stimulation (Fig 5B). α5 levels were shown to increase across all time points of nicotine stimulation, ranging from 1.5 to 2 fold (Fig 5B). Similar results were observed when cells were examined by immunofluorescence followed by confocal microscopy; α7 was increased upon nicotine stimulation for 48 hours (Fig 5C). To conduct time course experiments on protein levels, cells were rendered quiescent by serum starvation for 24 hours, and stimulated with 2μM nicotine for 18, 24, 48, 72, 96, or 120 hours. α7 protein levels were assessed using SDS-page western blot analysis or α7 mRNA expression was assessed by qRT-PCR. In A549 cells, α7 protein level increased beginning at 48 hours through 120 hours (Fig 5D); quantification of the blot is shown in the bottom panel. In H460s, α7 protein level increased beginning at 18 hours through 120 hours (Fig 5E), and the blot is quantified in the bottom panel.

**Fig 6:**
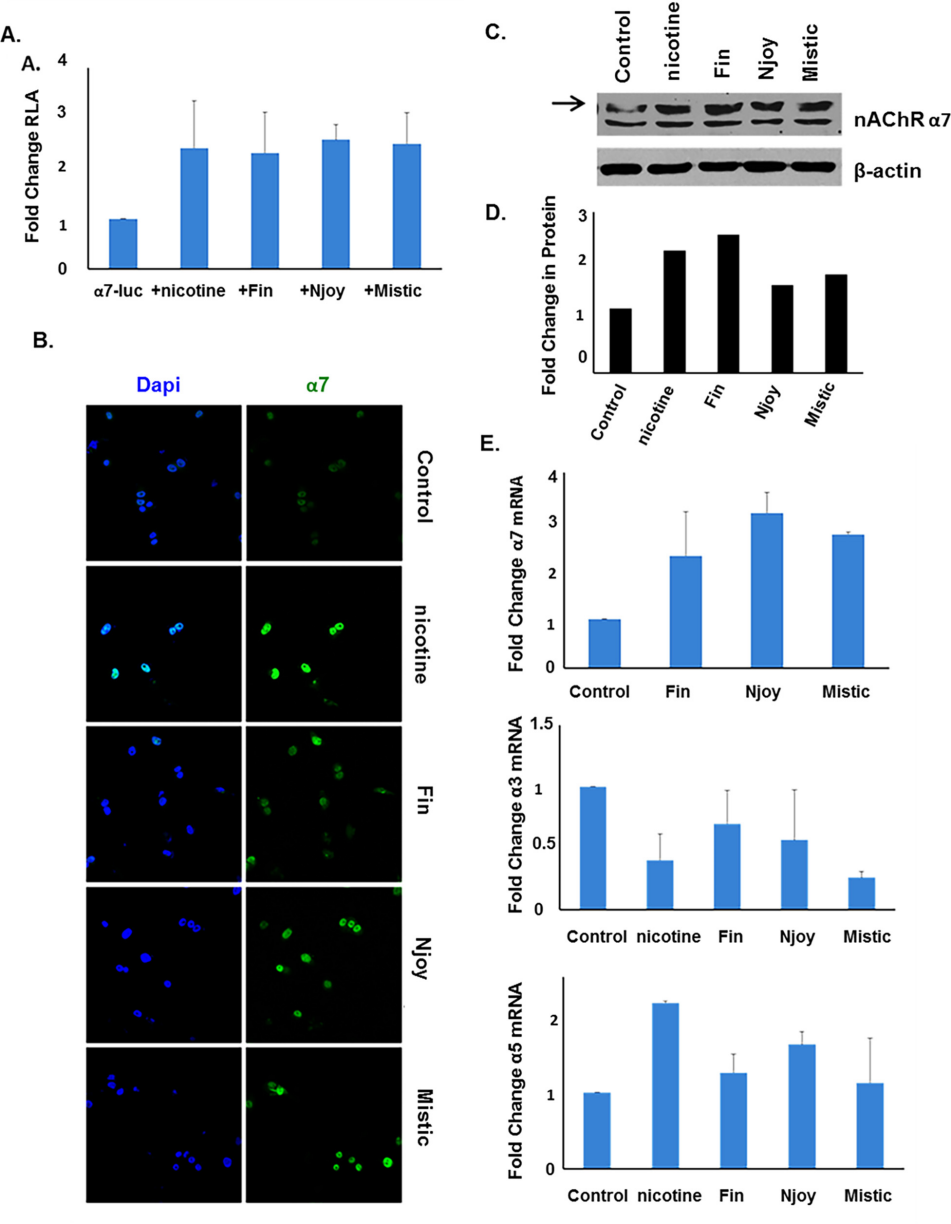
E-cigarettes can Induce α7 Expression at the Transcriptional, Translational, and Protein Levels. (A) Transient transfection showed that 1.5μM of three different brands of e-cigarettes could induce the α7 promoter after 48 hours of stimulation in A549 cells, to a similar extent as nicotine. (B and C) Immunofluorescent staining and western blot analysis showed that 1.5μM of e-cigarettes could induce α7 at the protein level after 48 hours in A549 cells, to a similar extent as nicotine. For western blot, arrow indicates the α7 protein band and quantification of the data is depicted in the corresponding graphs. (D) qRT-PCR analysis showed that 1.5μM of e-cigarettes resulted in increased α7 mRNA levels after 48 hours, decreased α3 mRNA levels after 24 hours, and increased α5 mRNA levels after 48 hours.

The health impact and implications of tobacco smoke and nicotine have been well documented; however, more recently electronic-cigarettes (e-cigarettes) have emerged as an alternative to cigarette smoking [45]. While e-cigarettes are advertised as less harmful tobacco-free alternatives that can also be used for smoking cessation, these products still contain nicotine in addition to other chemical compounds and their use is currently unregulated [46, 47]. Here we examined whether liquid extracts (containing nicotine) from three common brands of e-cigarettes Fin, Njoy and Mistic, had similar effects as nicotine on α7 nAChR expression. After 48 hours of stimulation with e-cigarette liquid, levels of α7 were assessed and were shown to increase at 1.5μM concentration. At the mRNA level, Fin induced α7 by 2.3 fold, Njoy induced α7 by 3.2 fold, and Mistic induced α7 by 2.7 fold (Fig 6D). Western blot analysis and immunofluorescent staining demonstrated that 1.5μM of each of e-cigarette could enhance α7 at the protein level (Fig 6B and 6C). Western blot results were quantitated and are depicted in Fig 6D. Transient transfection assays demonstrated 1.5μM of each e-cigarette to induce α7-luciferase promoter activity, as well (Fig 6A). To assess the impact of e-cigarettes on the other nAChRs implicated in lung cancer, qRT-PCR analysis was conducted. α3 mRNA levels were decreased after 48 hours of e-cigarette stimulation and α5 mRNA levels were increased after 48 hours of e-cigarette stimulation, both effects similar to that seen after 48 hours of nicotine stimulation (Fig 6D).

### Nicotine-mediated induction of α7 is abrogated by inhibition of Src, PI3K, MEK1/2, CDK4/6, Rb-Raf interaction, and α7 nAChR

**Fig 7:**
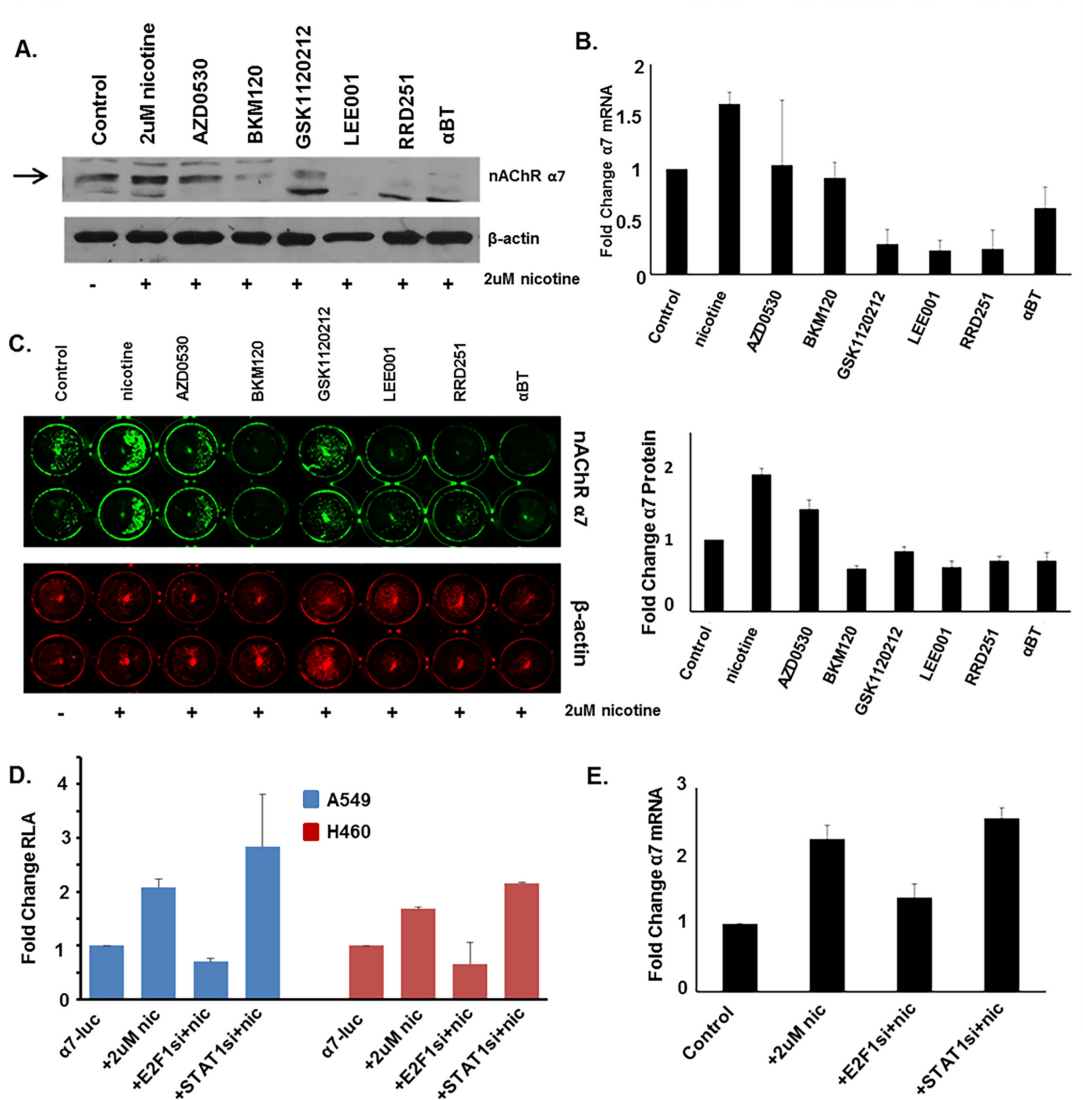
Nicotine-Mediated Induction of α7 Could be Abrogated by Inhibitors of Src, PI3K, MEK, CDK4/6, Rb/Raf, or α-BT; or by siRNA Depletion of E2F1. (A, B, C) Western blot, Li-Cor in-cell western blot, and qRT-PCR analysis showed that treatment with indicated inhibitors for 30 minutes prior to nicotine stimulation could abrogate the nicotine-mediated induction of α7 levels in A549 cells. For western blots, arrows indicate the α7 protein band. (D) Transient transfection showed that nicotine-mediated induction of the α7 promoter was decreased when E2F1 was depleted using siRNA, but was increased when STAT1 was depleted using siRNA in A549 and H460 cells. (E) Similar results were seen by qRT-PCR analysis which showed that nicotine-mediated induction of α7 mRNA was abrogated when E2F1 was depleted using siRNA, but enhanced when STAT1 was depleted using siRNA.

**Fig 8:**
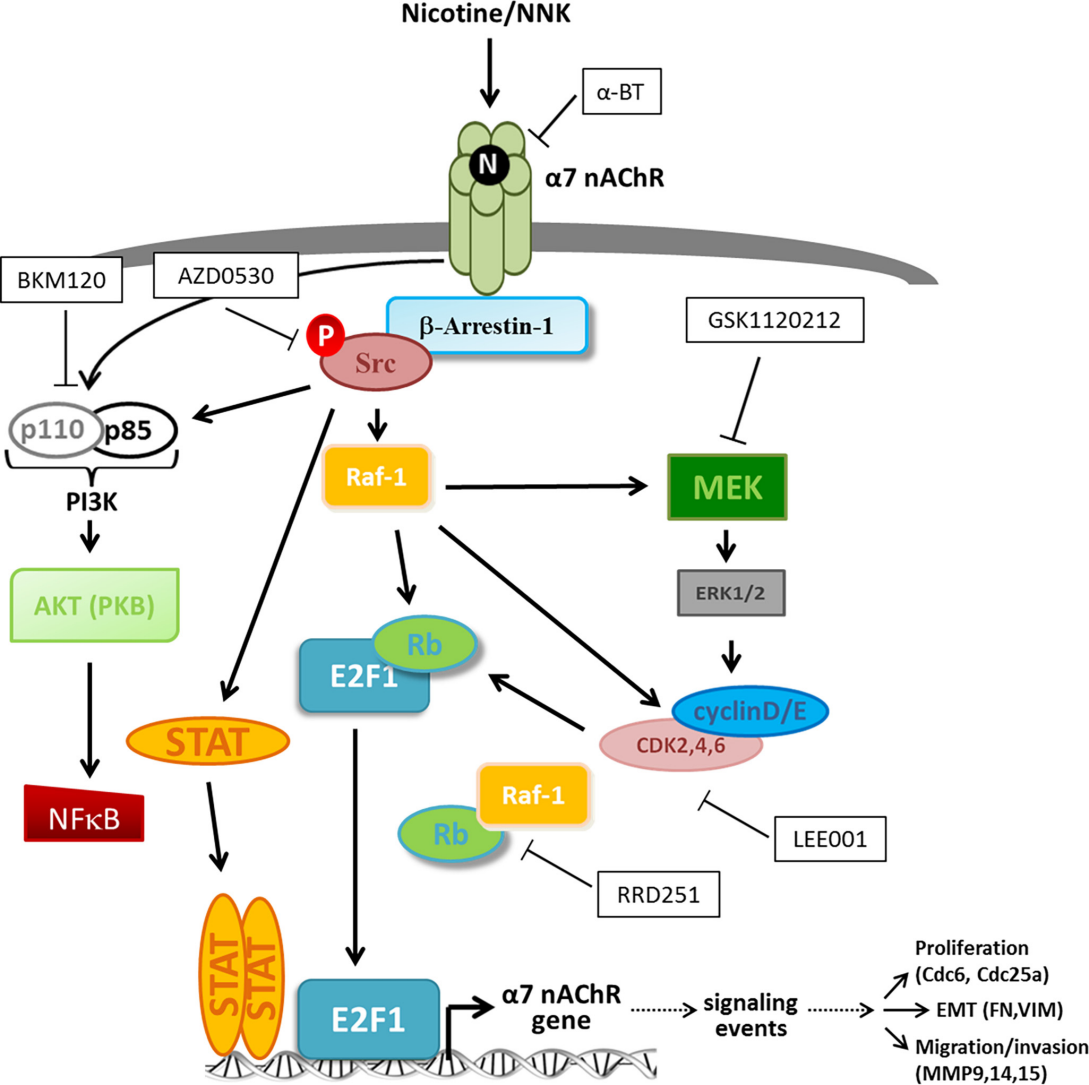
Schematic Depicting Signaling Cascades Initiated by Nicotine-Mediated Activation of the α7 nAChR and Inhibitors Used to Target these Pathways in this Study. Upon nicotine binding to α7 nAChR, oligomeric complexes form including the receptor, β-arrestin-1 scaffolding protein, and Src kinase. This activates Src, resulting in activation of Raf-1 kinase which acts along with activated CDK/cyclins to hyperphosphorylate the Rb tumor suppressor, resulting in its dissociation from E2F transcription factors, allowing them to activate their target genes including a number of genes involved in multiple aspects of tumor progression. The PI3K-AKT and MAPK signaling pathways are also known to be activated by nicotine-mediated α7 nAChR activation subsequent to Src phosphorylation; and activation of MEK-ERK is known to result in activation of CDK/cyclin complexes. AKT1 activation downstream of PI3KC in response to nicotine has additionally been shown to result in activation of NFκB transcription factors. Further, Src activates STAT proteins up nicotine-mediated activation of α7 nAChR. Inhibitors used in this study are depicted in black and white boxes, and their targets are indicated accordingly.

To elucidate the mechanism by which nicotine induces α7 expression, experiments were conducted using inhibitors to signaling pathways that are activated in response to nicotine stimulation. Previous studies have identified that Src kinase is activated upon nicotine stimulation and subsequently activates downstream kinases including PI3K and Raf-1 [11, 21]. PI3K is classically known to activate AKT/PKB, followed by activation of transcription factors such as NFκB, which initiates transcription of a number of tumor promoting genes. Similarly upon nAChR activation, Raf-1 and cyclin/CDKs phosphorylate Rb tumor suppressor protein, resulting in the induction of E2F-mediated transcription of proliferative genes. Studies have also shown that nicotine stimulation activates the MAPK cascade. To elucidate whether these pathways are involved in nicotine-mediated induction of α7 expression, cells were stimulated with nicotine in the presence or absence of the following inhibitiors: AZD0530/Saracatinib targeting Src, BKM120/Buparlisib targeting α, β, δ, and γ isoforms of PI3K, GSK1120212/Trametinib targeting MEK1/2, LEE001/Ribociclib targeting CDK4/6, RRD251 targeting Rb-Raf-1 interaction, and α-bungarotoxin/α-BT, which is an α7 nAChR antagonist. Western blot analysis demonstrated all six inhibitors abrogated nicotine-mediate induction of α7 protein levels, although the Src inhibitor AZD had a modest effect (Fig 7A). These results were confirmed by in-cell western blotting which quantitates protein levels. 48 hours of nicotine stimulation induced α7 expression 2 fold, while treatment with AZD, BKM, GSK, LEE, RRD, or α-BT 30 minutes prior to nicotine stimulation reduced α7 levels by 67%, 32%, 45%, 34%, 34%, and 40%, respectively (Fig 7C). These results were additionally confirmed at the mRNA level by qRT-PCR, which showed nicotine to induce α7 mRNA levels by 1.6 fold, while AZD, BKM, GSK, LEE, RRD, or α-BT reduced α7 levels by 61%, 58%, 18%, 14%, 15%, and 39%, respectively (Fig 7B). This data suggests that the pathways or proteins targeted by these inhibitors play a role in nicotine-mediated induction of α7 nAChR, as detailed in the schematic presented in Fig 8, and if impeded by inhibition result in abrogation of nicotine-mediated induction of α7.

### E2F1 is necessary for nicotine-mediated induction of α7

Since we find that E2F1 and STAT1 regulate α7 nAChR expression, we sought to determine whether the effect of nicotine on α7 occurs through E2F1 and STAT1. To test this, cells were transfected with small interfering RNA (siRNA) to deplete E2F1 or STAT1, and subsequently stimulated for with 2μM of nicotine for 48 hours, and α7 levels were assessed. We found that when E2F1 was depleted there was diminished ability of nicotine to induce α7, and when STAT1 was depleted nicotine further induced α7-luciferase or α7 at the protein or mRNA level in A549s (Fig 7D and 7E). At the RNA level, nicotine induced α7 by 2.3 fold, which was reduced to by 61% upon E2F1 depletion, and increased by 13% upon STAT1 depletion (Fig 7E). This was further confirmed when 1μg of α7-luciferase was transfected into cells and was shown to increase 2.1 fold with 48 hours of nicotine stimulation, an effect which was reduced by 33% upon E2F1 depletion and increased by 33% upon STAT1 depletion in A549; similarly, in H460s nicotine increased α7-luciferase 1.7 fold, which was reduced by 41% upon E2F1 depletion, and increased by 29% upon STAT1 depletion (Fig 7D).

## Discussion

Cigarette smoking is highly correlated with NSCLC and accounts for 80–90% of all lung cancer deaths [3]. Our lab and others have previously reported that nicotine, which is the addictive component of tobacco smoke, while not thought to initiate tumors itself can enhance a number of tumor promoting properties including proliferation, migration, invasion, epithelial-to-mesenchymal transition (EMT), and angiogenesis [11, 21, 35, 48, 49]. Further, nicotine is known to mediate therapeutic resistance, survival/resistance to apoptosis, self-renewal of cancer stem-like cells, as well as modulate a number of immune properties in cancer [12, 50–52]. These tumor promoting effects have been shown to occur primarily through the binding to and activation of nAChRs; and proliferation, migration, invasion, and EMT have been shown to occur through the α7 nAChR subunit in specific, implicating it in tumor progression. The α7 nAChR subunit is overexpressed on human NSCLC tumors compared to normal adjacent, and nicotine can enhance expression of the α7 receptor itself, further demonstrating the importance of α7 in nicotine-mediated pathophysiological effects; however, how this occurs and how the α7 nAChR gene is regulated is less understood.

In squamous cell carcinoma of the lung (a subset of NSCLC), it has been previously reported that nicotine increases α7 nAChR expression which accelerates tumor growth and results in worse clinical outcomes [33]. In this context, nicotine increased α7 expression through transcriptional mechanisms involving Sp1 and GATA4/6 proteins. Nicotine enhances association of these proteins to the promoter, and they are required for its nicotine-mediated induction. The transcriptional regulation of the α7 nAChR promoter in the context of adenocarcinoma of the lung, as well as the mechanisms by which this occurs, still remains elusive. In this study we demonstrate that E2F1 and STAT1 transcription factors act to differentially regulate the α7 gene promoter and disruption of STAT binding sites can diminish this effect. Further, we find that nicotine-mediated induction of α7 can be abrogated by inhibitors to Src, PI3K, MEK1/2, CDK4/6, Rb-Raf-1 interaction, or α7 nAChR suggesting the pathways targeted by these molecules play a role in nicotine-mediated induction of α7 and their disruption inhibits this. As depicted in Fig 8, stimulation of cells with nicotine activates Src via β-arrestin-1, facilitating the downstream signaling events. These signaling events lead to the enhanced expression of α7 nAChR, which in turn amplifies the same signaling cascades. Our earlier studies had shown that signaling via α7 nAChR leads to the expression of genes involved in cell proliferation, like Cdc25, thymidylate synthase and Cdc6 [34]; those involved in epithelial-mesenchymal transition like ZEB-1 and ZEB2 as well as fibronectin and vimentin [7, 35, 53] and genes involved in invasion and metastasis, including multiple matrix metalloproteinases [11, 54]. Overall, it is highly likely that cells exposed to nicotine for extended periods of time will overexpress α7 nAChR, which we had observed in a mouse model [17]; this will lead to the induction of genes involved in tumor progression and metastasis, thus facilitating the tumor promoting functions of nicotine.

Interestingly, we also demonstrate that increasing amount of one transcription factor could abrogate the effect of the other on α7 promoter expression, suggesting a competitive interplay between E2F1 and STAT1. A number of previous studies have reported similar findings of competition between transcription factors to differentially regulate gene expression, across various contexts. For example, in macrophages it has been shown that AP-1, STAT1, and STAT3 compete with NFκB to bind a gene encoding aralkylamine N-acetyltransferase and differentially regulate its expression to control the synthesis of melatonin and inflammatory response [55]. In colorectal cancers it has been shown that β-catenin/TCF4 complexes compete with TCF3 to bind to the MYC promoter, and β-catenin/TCF4 induces its expression while TCF3 represses it [56]. HNF4α has also been found to compete with TCF4 and AP-1 at overlapping motifs to regulate distinct genes involved in proliferation and inflammation in colorectal cancer [57]. Notably, a number of reports of competitive transcription factor interplay involve TCF, AP, SP-1, and STAT transcription factors [55–59]. While this suggests that such a dynamic to differentially regulate gene expression may be a common mechanism occurring in response to environmental stimuli, this is the first demonstration of such a mechanism involved in the regulation of nAChR expression.

Recently, the use of e-cigarettes has emerged as an alternative to traditional cigarette smoking and as a smoking cessation agent. These nicotine-delivery devices represent a public health concern, and mounting evidence suggests that the liquid containing nicotine found within the cartridge of e-cigarettes, which is delivered as a vapor/aerosol to the user, elicits detrimental health effects similar to those of nicotine from traditional cigarettes [60]. There has been an increase in their use among both youths and adults [61]. Typically, e-cigarettes consist of a battery, a heating element, an air flow sensor, and a cartridge containing liquid which includes nicotine in addition to other components such as propylene glycol, glycerol, and other additives. When activated the liquid is heated and is delivered as an aerosol in a vapor form to the user [60]. Each brand varies in concentration of nicotine present in the liquid, but it is typically indicated as percent nicotine by volume (NBV) on the packaging of the item. While e-cigarettes are thought to be better than traditional cigarettes since they lack tobacco which is known to contain multiple classes of carcinogens, the presence of nicotine may still largely impact pathophysiology and health in users. In this study, we report that e-cigarettes could induce α7 nAChR levels in a manner similar to nicotine. Further research is underway to elucidate whether these products can also enhance other tumor-promoting properties, and whether this occurs through the same mechanisms as nicotine-mediated tumor progression.

Of interest, our Kaplan Meier analysis revealed that high levels of the CHRNA7 gene encoding α7 nAChR correlated with increased patient survival in NSCLC, albeit the known role of α7 to facilitate tumor progression. It is unclear why this is; however, we can speculate that there may be a number of factors at play. First, it is possible that tumors may have higher levels of α7 in early stages of tumorigenesis (while the tumor has better prognosis) which would enhance the ability of the tumor to progress, but as the tumor advances to late stages α7 levels decrease and other factors are driving tumor progression. Another possibility is that patients with higher levels of α7 may respond better to treatment modalities for undefined reasons, resulting in increased patient survival despite high levels of this receptor; while α3/β2 receptor subunits were involved in conferring resistance to chemotherapy drugs, α7 did not appear to have a role [62]. It could also be that when there are higher levels of α7 present in a tumor, the receptors become desensitized to stimulation and are no longer able to be activated to exert their biological function; or alternatively they could become hyperactivated resulting in desensitization in a manner similar to the tumor inhibitory nAChR α4β2 which becomes desensitized in response to constant high affinity stimulation [63]. Further studies are warranted to answer this question.

The elucidation of regulation of nAChRs involved in nicotine-mediated cancer progression and the signaling mechanisms involved presents new opportunities to develop agents targeting these pathways, which could potentially improve therapeutic outcome for patients with smoking-related cancers. This study helps us to better understand the pathophysiology of such disease, specifically in the context of nicotine-mediated promotion of NSCLC.
